# Perceptions on Adherence to Dietary Prescriptions for Adults with Chronic Kidney Disease on Hemodialysis: A Qualitative Study

**DOI:** 10.3390/diseases8030029

**Published:** 2020-08-06

**Authors:** Rose Okoyo Opiyo, Susan Akoth Nyawade, Michael McCaul, Peter Suwirakwenda Nyasulu, Daniel Bolo Lango, Anthony Jude Omolo Were, Esther Clyde Nabakwe, Zipporah Nekesa Bukania, Joyce Muhenge Olenja

**Affiliations:** 1 Disease Prevention, Control and Health Promotion Unit, School of Public Health, College of Health Sciences, University of Nairobi, P.O. Box 19676-KNH, Nairobi 00202, Kenya; wuodojema@gmail.com; 2 East African Kidney Institute, College of Health Sciences, University of Nairobi, P.O. Box 19676-KNH, Nairobi 00202, Kenya; drajowere@gmail.com; 3 Community Health Sciences Unit, School of Public Health, College of Health Sciences, University of Nairobi, P.O. Box 19676-KNH, Nairobi 00202, Kenya; susanakoth2010@gmail.com (S.A.N.); jolenja@uonbi.ac.ke (J.M.O.); 4 Division of Epidemiology and Biostatistics, Department of Global Health, Faculty of Medicine and Health Sciences, Stellenbosch University, Tygerberg, Parow, Cape Town 7505, South Africa; mmccaul@sun.ac.za (M.M.); pnyasulu@sun.ac.za (P.S.N.); 5 Department of Internal Medicine, College of Health Sciences, University of Nairobi, P.O. Box 19676-KNH, Nairobi 00202, Kenya; 6 Department of Child Health and Pediatrics Moi University, P.O. Box 4606, Eldoret 30100, Kenya; nabakwe@cartafrica.org; 7 Centre for Public Health Research—Kenya Medical Research Institute, P.O. Box 20752, Nairobi 00202, Kenya; zbukania@gmail.com

**Keywords:** perceptions, adherence, nutrition, renal, Kenya, dialysis, food, kidney

## Abstract

Diet is one of the modifiable lifestyle factors in management of kidney disease. We explored perceptions on adherence to dietary prescriptions for adults with chronic kidney disease on hemodialysis. This was a qualitative descriptive study. Participants were purposively selected at renal clinics/dialysis units at national referral hospitals in Kenya. Data were collected using in-depth interviews, note-taking and voice-recording. The data were managed and analyzed thematically in NVIV0-12 computer software. Study participants were 52 patients and 40 family caregivers (42 males and 50 females) aged 20 to 69 years. Six sub-themes emerged in this study: “perceived health benefits”; “ease in implementing prescribed diets”; “cost of prescribed renal diets”; “nutrition information and messages”; “transition to new diets” and “fear of complications/severity of disease”. Both patients and caregivers acknowledged the health benefits of adherence to diet prescriptions. However, there are mixed messages to the patients and caregivers who have challenges with management and acceptability of the prescriptions. Most of them make un-informed dietary decisions that lead to consumption of unhealthy foods with negative outcomes such as metabolic waste accumulation in the patients’ bodies negating the effects of dialysis and undermining the efforts of healthcare system in management of patients with chronic kidney disease.

## 1. Introduction

According to the most recent data on global burden of disease, in 2017, about 1.23 million people died from chronic kidney disease (CKD) globally and close to 5000 of the deaths were from Kenya [1]. Kenya has seen an increase in the number of diagnosed cases of CKD annually. It is further estimated that 4.8 million Kenyans will be suffering from kidney disease by 2030 [2]. Consequently, the number of patients undergoing chronic hemodialysis in Kenya also increased from 300 in the year 2006 to 2400 in 2018 [3] due to the increase in availability of kidney services, particularly the doctors/nephrologists, renal nurses and hemodialysis units in most parts of the country since 2015 [4]. National data on mortality of hemodialysis patients is not accessible for most countries including Kenya. However, the Kenya Renal Association’s dialysis registry indicate that during the period of August 2018 to July 2019, 11 deaths occurred among 136 hemodialysis patients at Kenyatta National Teaching and Referral Hospital, reflecting a case fatality rate of 8.0%. Whereas other pre-existing co-morbidities may be responsible for mortality of patients on hemodialysis, diet-related factors have also been shown to be a contributory factor [5,6]. 

Although nutrition is one of the modifiable lifestyle factors in management of CKD [7,8,9] adherence to dietary prescriptions is a challenge for more than half of adult patients globally [10,11]. In Kenya, the only study on adherence to diet prescriptions also indicate that more than half of adults on hemodialysis at national referral hospitals do not adhere to their diet prescriptions [12]. For the patients with CKD on hemodialysis, Medical Nutrition Therapy entails dietary restrictions to control accumulation of metabolic wastes and development of comorbidities [13,14]. Yet, many patients with CKD consider the renal diets to be contradicting routine and recommended diets for healthy individuals [15]. Furthermore, the variations in these diets due to stage of the kidney disease, comorbidities and type of treatment is often confusing and contradictory for the patients to comprehend [15,16]. According to the health belief model [17], people will be more motivated to modify their behavior if they believe they are susceptible to a particular negative health outcome with severe consequences. Research suggests that in CKD, the stronger the perception of severity of the negative outcome, the more the patients will be motivated to take actions to prevent its occurrence [18]. Whereas the available research evidence on diet and CKD prevention may not be convincing, it is possible that adherence to diet prescriptions may prevent complications associated with the disease and contribute to improvement in patients’ quality of life [14,19]. Research findings from Kenya indicate that diet prescriptions with foods that are considered less restrictive are more likely to be adhered to than the ones that do not match with the patient’s other ways of eating as well as food preparation methods [12]. These perceptions, in the context of the multi-dimensional nature of factors, including poor motivation, contribute to non-adherence to therapies [20,21] and are more likely to exacerbate the non-adherence to dietary prescriptions and contribute to rapid progression of CKD among patients. A contextual understanding of the patients’ perceptions towards renal diets is therefore important for successful dietary management of CKD. There are limited studies on perceptions to dietary restrictions among adults with CKD from African countries [22,23,24] where regional variations exist in the health care systems as well as household food security that shape individual’s perceptions on adherence to prescribed diets. In Kenya, there is minimal documented evidence on adherence to CKD diet prescriptions [12]. 

The purpose of this study was to explore the perceptions about adherence to dietary prescription among adults with CKD on hemodialysis and their family caregivers. This is expected to contribute to the knowledge-base and evidence for slowing down progression of kidney disease in adults on hemodialysis based on caregivers’ and patients’ own perceptions.

## 2. Materials and Methods

### 2.1. Study Design and Setting

This was a qualitative descriptive study. The methods and results have been reported in accordance with the consolidated criteria for reporting qualitative research (COREQ) [25]. The study was conducted at the renal clinics and dialysis units at two sites: Kenyatta National Hospital (KNH) and Moi Teaching and Referral Hospital (MTRH) in Kenya. The KNH is located in Nairobi, the capital and largest city of Kenya while MTRH is located in Eldoret Town, north western part of Kenya. These two referral hospitals were selected due to the large numbers and diversity of patients attending the renal clinics from various regions, communities and cultures in Kenya. The total number of patients receiving dialysis per month during the study period was about 100 at KNH and 70 at MTRH. 

### 2.2. Study Population and Selection

The study participants included adult patients, 18 years and above, with CKD on hemodialysis and their family caregivers who lived with them and were normally responsible for their meal preparation and feeding-related activities. They were purposively selected at the renal clinics/dialysis units based on the inclusion/exclusion criteria. Only patients in stable health condition, able to communicate well at the time of data collection and willing to provide written consent participated in the study. Excluded were patients with kidney transplant, not in stable condition, unable to respond or not accompanied by a caregiver. Study participants were consecutively recruited from the group of all adult patients with CKD on hemodialysis therapy. For caregivers, inclusion required that they be adult household members, 18 years and above, accompanied the CKD patient to the hospital and provided a voluntary informed written consent. The caregivers whose patients were selected were excluded to ensure that we obtained a variety of perceptions from different households and avoid duplication of information. The selection of participants was determined by the saturation point when no new information emanated from the transcribed interviews while ensuring diversity in representation of participants’ characteristics. 

### 2.3. Data Collection Instruments 

Data were collected using in-depth interview guides with open-ended questions and digital audio recorders. The design of the data collection instruments was informed by the research question and the health belief model [17] as well as the “patients’ perceptions of renal dietary advice tool” [15]. The questions for the participating patients covered awareness of renal diets, sources of nutrition information, experiences, perceptions on adhering to the diets as well as motivating factors and challenges to adherence. For the caregivers, the interviews focused on their awareness of the diet for the CKD patients, their perceptions on the recommended diets and whether their patients adhere to the diets as well as their observations and experiences during the period from ordinary to prescribed diets. A checklist of key prompts on patient-related, condition-related, socio-economic-related, therapy-related and healthcare-related factors [20,21] was used alongside the open-ended questions to ensure comprehensive discussions on perceptions and consistency in probing among the interviewers. 

### 2.4. Research Process

**Figure 1:**
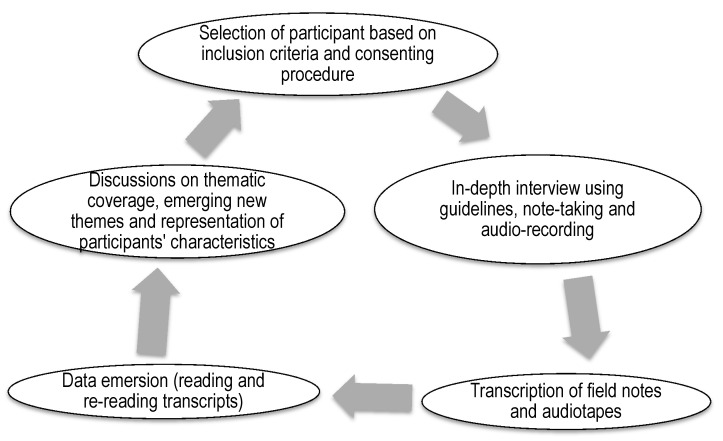
Summary of the research process: data collection and investigation process involved repetitive interactions between participants, field research teams, principal investigator and the person coding the data.

The in-depth interview guides and audio-recorders were pre-tested among a small group with similar characteristics at the renal clinics and dialysis wards at each of the study hospitals. The purpose of the pre-test exercise was to identify practical problems with regard to data collection tools, session duration, and methodology [26]. Participants in the pre-test did not participate in the main study. Details of the study were explained to each participant prior to conducting the in-depth interviews and those who were willing to participate signed the consent form. Each interviewer selected a participant based on inclusion criteria and obtained consent from the participant to conduct the in-depth interview. The interviews lasted for 30 to 60 min. All the interviews were recorded by both note-taking and voice-recording on a universal serial bus (USB) audio-recorder. An iterative approach involving repetitive interactions between the participants, field research teams, principal investigator and the person coding the data was adopted in data collection and investigation process (Figure 1). Records of the interviews were shared with the data monitor, a senior qualitative researcher, for content check, and the principal investigator (PI) for content confirmation and formatting of the typed documents before data coding by an independent research team member. The PI consolidated the feedback from the data monitor and coder on the interviewers’ performance and shared during daily de-briefing sessions. Regular discussions, at least once every week, between the PI, monitor, coder and interviewers were conducted to compare the information emerging from the interviews and the main thematic coverage, emerging themes, socio-demographic and co-morbidity representation of the data collected. 

### 2.5. Trustworthiness of the Study 

Trustworthiness in qualitative research refers to credibility, transferability, dependability and confirmability of the research process [27]. In this study, credibility was ensured through several ways. First, triangulation of the data occurred at various levels: site selection, categories of participants and recording of the interviews. The two study sites ensured representation of data from diverse regions and communities in Kenya. The data collection from the two categories of participants ensured that we obtained the perspectives from two key players in the managements of CKD diet prescriptions. Triangulation of the data through the use of voice recording and note taking ensured that all the participants’ information was captured in the transcripts further contributing to the credibility of the study findings. Second, credibility was ensured as the researchers developed trust and rapport with participants by creating a comfortable and trusting environment for them to freely participate in the interviews. Third, credibility was ensured through adoption of existing data collection tools from literature on patients’ perceptions to renal diets [15] as well as a checklist of key prompts on factors affecting adherence [20,21]. Fourth, subsequent interviews were also varied where necessary to enhance the depth of information gathered. Fifth, credibility of the data collection process was further ensured through monitoring of the data collection by a senior qualitative researcher who regularly checked on the data collection teams in addition to reading their scripts and providing the interviewers with timely advice on the data collection process. 

Transferability of the study findings was ensured through detailed documentation of the research process (Figure 1) and participants characteristics. The study participants’ contexts in terms of gender, age, co-morbidities and type of participant (patient or caregiver) were collected, recorded in the individual transcripts, analyzed and presented as introduction to findings. This allows the study findings to be applicable to other patients with similar characteristics. The transferability is however limited to adults with CKD on hemodialysis within the renal unit environment in a hospital setting. 

The detailed description and summary of the research process illustrated in Figure 1 also allows for dependability and confirmability of this study. Confirmability was further ensured through reflexivity where the research team members who included the PI, research assistants and the data coder assessed the data independently in order to minimize their personal biases. 

### 2.6. Recruitment and Training of Interviewers

A total of five interviewers, two stationed at KNH, and three at MTRH were involved in the data collection process. They were recruited based on set criteria that included at least a Bachelors’ Degree in Nutrition or Public Health and past experience collecting qualitative data. In addition, each site had a data monitor to oversee the data collection process and give timely advice to the interviewers. Two male and three female research assistants conducted the interviews to ensure gender balance to minimize bias in the data collected. 

All the interviewers were trained on the research process with emphasis on background and objectives of the study, how to conduct the in-depth interviews, use of research interview guides, recording and transcribing the interviews and ethical issues to be observed during the study, particularly the rights of participants and obtaining their consent to participate in the research.

### 2.7. Data Management and Analysis 

Iterative analysis was conducted on thematic area of perceptions. This started during the data collection when the PI held frequent meetings with the field teams and the data coder to discuss coverage and nuances of these themes. Thematic descriptive analysis was then conducted, based on existing theme decided a priori on perceptions to identify emerging related (sub)-themes. The NVIVO 12 computer software (Version 12, QSR International, LLC, 35 Corporate Drive, Burlington, MA, USA) was used to aid in thematic analysis of the data. The number of times a theme appeared was considered to determine the categories of themes created as well as any similarities and differences between and within region and participant type. 

## 3. Findings

### 3.1. Description of Study Participants 

A total of 92 participants (patients = 52; caregivers = 40) were interviewed in this study. More than half of participants were females (50 out of 92). Participants’ ages ranged from 20 to 69 years, and more than half of them, (59 out of 92), were within the age of 40 years and above. The most common co-morbidity among the patients with CKD was hypertension. Awareness of dietary restrictions for patients with kidney disease was high (86 out of 92).

A majority of patients (29 out of 52) reported positive social relationship with their caregivers and family members who gave them unqualified support as summarized in the following quote from one of the participants:

“They give me support, like my sister, she is the one who has been supporting me a lot. When I began that diet, and she is the one who prepares most meals at home. When she cooks, she makes sure that mine is mine, and their food is theirs” (Female Patient, 31, Uasin Gishu County).

Most caregivers were spouses (*n* = 15) and off-springs (*n* = 12) of the patients. There were also six parents who were taking care of their children and four siblings taking care of their brothers/sisters. Almost all participants were aware of the recommended diets. They reported that they acquired the information from the health facility after they were diagnosed with CKD as evidenced in the following verbatim statements:

“The fruits, which I have been advised to take, are papaws which are ripe because they have very little acid content, then also mangoes which are equally very ripe and watermelon. I am advised not to consume the seeds of watermelon, just to take the fruits” (Male Patient, 56, Kakamega).

### 3.2. Main Findings on Perceptions on Adherence to Dietary Prescription

Six sub-themes on perceptions on adherence to dietary prescription emerged from this study: perceived health benefits (reported by all participants); ease in implementing the prescribed diets (reported by all participants); cost of prescribed renal diets (reported by 21 patients and 20 caregivers); nutrition information and messages (reported by 16 patients and 12 caregivers); transition to new diets (reported by 9 patients and 13 caregivers) and fear of health complications and severity of disease in case of non-adherence to the prescribed diets (reported by 4 patients and 7 caregivers). The emerging issues under each of these sub-themes are presented in the next sub-sections with illustrative quotes presented in the sub-sequent tables. 

### 3.2.1. Sub-Theme 1: Perceived Health Benefits 

All participants made judgment on the renal diet recommendations based on the perceived health benefits of the food. Some of the verbatim statements from participants on health benefits of adherence to the diet prescriptions are presented in Table 1. Apparently, participants who felt that they understood the health benefits of strictly following diet recommendations were optimistic about their health. In their view, patients with CKD felt better upon adhering to prescribed diet, but suffered if they did not adhere. Some participants felt that patients were likely to live longer (and hopefully have a better quality life), if they followed the dietary prescriptions they from healthcare workers. Others also felt encouraged to follow the recommended diets after discovering that other patients who followed the same diets had improvements in their health and quality of life. As for the patients who did not adhere to their diet prescriptions, however, it was noted that perceived negative health effects were apparent.

### 3.2.2. Sub-Theme 2: Ease in Implementing Prescribed Diet 

Participants perceived the recommended diets as challenging to implement as illustrated by the verbatim quotes in Table 2. The cooking of food separately was considered a major challenge in the context of limited resources particularly time, cooking utensils and pots as well as fuel. This was due to the differences in cooking methods such as frying food for family members vis-a-vis boiling for the patient or cooking green vegetables for family members vis-a-vis cooking cabbage for the patient. The different cooking and preparation methods required extra time and resource inputs. For some patients, eating out in social gatherings also became very difficult because of the need to keep explaining to people the reasons for avoiding certain foods. This affected their social life as they resorted to withdrawing from social gatherings. Where household members adapted to eating the food recommended for the patients with CKD, implementation of the prescribed diets for the patients was not a challenge.

### 3.2.3. Sub-Theme 3: Cost of Prescribed Renal Diets

Close to half of all participants (42 out of 92) perceived the prescribed foods as expensive and not affordable. These sentiments were echoed by 21 patients and 21 caregivers. Not only did participants find it expensive to buy the specific prescribed fruits, vegetables and white meat, but some reported that they could not afford the required daily meal frequencies. Some of the recommended fruits were also not locally available, hence expensive. However, those who reported reliable sources of income did not perceive the diets to be expensive. For them, the prescribed renal diets were too restrictive rather than expensive (Table 3).

### 3.2.4. Sub-Theme 4: Nutritional Information and Messages

Participants interviewed mostly reported that they understood the nutrition counseling message they received from the health facility. However, the verbatim statements in Table 4 indicate that they perceived the nutrition information from the health facility as inadequate and sometimes confusing for them. They argued that sometimes the nutritionist and nurses gave different messages which seemed contradictory. They also complained about lack of consistency in nutrition information from different nutritionists at the health facility. Some patients, therefore, seemed confused about the diet prescriptions and opted to ignore the nutritional advice or triangulated the information with their fellow patients to know the best food to eat. However, for some patients, the messages from the nutritionist were clearly understood and they followed what they had been told to eat. 

### 3.2.5. Sub-Theme 5: Transition to New Diets 

Upon diagnosis with kidney disease, patients have to forego certain items in their diet such as table salt and getting used to these new diets poses a challenge for many. In this study, those who understood the health benefits of eating the prescribed food had no problem in adjusting to the new diets. However, majority of both caregivers and patients perceived the transition to prescribed diet as challenging and not easy to get used to. It is also a common belief that during food preparation, salt must be added to food for it to taste. Many patients, therefore, found it difficult to eat food without salt. Their first reaction when they were told about the new diet was anger, denial and refusal to take the prescribed diets. However, for those who perceived these diets as beneficial for their health, transition to the new diets was easy (Table 5). 

### 3.2.6. Sub-Theme 6: Fear of Health Complications and Severity of Disease 

Our findings indicate that some patients and caregivers fear that health complications and perhaps an increased severity of disease may arise from non-adherence to diet prescriptions. Some caregivers’ arguments on their perceptions about the severity of CKD were related to the changes they observed in their patients’ condition following non-adherence. These changes included lack of appetite, vomiting, weight loss and stomach aches. Data showed that patients were aware of their susceptibility to complications, especially if they did not adhere to the recommended diets and thus perceived most of the health complications emanated from consumption of the restricted foods (Table 6). 

## 4. Discussion

### 4.1. Discussion of Key Findings

In this study, we explored the perceptions on adherence to dietary prescription among adults with CKD on hemodialysis and their family caregivers. The findings hinged on six critical sub-themes: “perceived health benefits”, “ease in implementing the prescribed diets”, “cost of prescribed renal diets”, “nutrition information and messages”, “transition to new diets” and “fear of health complications and severity of the disease”. These will be discussed in detail with emphasis on their relevance in terms of management of patients with CKD and the caregivers’ role in providing relevant and adequate support. 

Adherence to diet prescription is one of the modifiable lifestyle factors in the management of CKD [7,8] and our findings demonstrated that it is perceived to provide a positive health benefit [17] and quality of life. This suggests that potentially, it can be easily adopted by both patients and caregivers. The perception of the renal diets “good for health and kidneys” as a health benefit by all participants suggested that it was a motivation to adhere to the diets. However, adherence to diet prescription among this patient population in Kenya is low despite awareness of the dietary restriction [12]. It is, therefore, possible that study participants made judgment on the renal diet recommendations based on their perceived health benefits of the food. These findings intimated that patients understood the health benefits of strictly following diet recommendations and were optimistic about better quality of life. They expressed how they felt better upon adhering to renal diet, but suffered if they did not. The experiences of patients who “feel better” when they adhere to the diet prescription, but “suffer” due to non-adherence are worth sharing widely with other patients who struggle with dietary management of their health conditions. Similar findings where patients with CKD on hemodialysis have attributed improvement of their health conditions to adherence to diet prescriptions have been previously reported in other studies [28,29]. It is possible that patients regard adherence to diet prescription as an important aspect of their hemodialysis treatment that provides tangible benefits to their health as posited in the health belief model where a target behavior must provide a strong positive health benefit for it to be adopted [17]. It was, therefore, not surprising that, in our study, fear of health complications when the recommended diet was not followed appeared as a motivating factor in adherence. The perception of severity of the negative health outcome has been known to motivate patients to take actions to prevent the occurrence of the health problem [17]. In our study, patients understood that it was partly their responsibility to decide the period and the quality of life they wished to live in the context of their condition by playing the card of adherence. The positive impact of perceived illness threat and consequences of non-adherence to diet has also been reported in other domains of chronic conditions’ management [30].

Whereas all participants perceived adherence to diet prescriptions as beneficial for their health, all of them also perceived these diets to be challenging and difficult to implement. The diet restrictions are viewed as complex and at times contradictory to perceived healthy eating, cultural norms, social functioning, and individuals’ sense of control [16]. The prescribed diets are considered stressful and expensive because the foods and cooking methods are different from the household members’ usual meal requirements as well as difficult and frustrating to integrate into usual diet and eating habits [12,31]. Similar sentiments were also expressed by Chironda and Bhengu [20] who concur that CKD and its treatments can indeed restrict daily activities, employment, family life, and social relationships, often leading to feelings of isolation that might induce stress and anxiety. Cultural eating habits have however been known to preserve social identity and participation, maintain social status, and reduce the stigma of being ill [28]. Our participants reported weighing adherence to these dietary restrictions against social effects on their quality of life. In this regard, patients may be willing to accept a reduced life expectancy in exchange for fewer restrictions in their social life. Interventions that minimize the effect of these restrictions should, therefore, form an integral part of care [31]. Participants also considered the diet prescription as concepts that were externally imposed on them. They preferred to be allowed to choose what to eat. Their views about having life’s pleasures removed and food being tasteless demonstrated how unhappy they were with adhering to the diet prescription. However, with positive social support from family caregivers and friends, adjusting to the renal diet could be easier. Social support has been shown in existing literature to improve survival and quality of life in dialysis patients by dispelling hopelessness, providing a morale boost thereby positively influencing their adherence to both medication and dietary recommendations [29,32]. The concerted effort and emotional/moral support from family members help patients to make the necessary adjustments in the reality of the disease and dialysis [33]. 

Poor socio-economic status can contribute to inadequate access to prescribed diets, hence, predispose patients to non-adherence. As reflected by current findings, economic challenges and cost of diets have previously been reported to limit dietary options of individuals [20,34,35,36,37,38,39] This exacerbates the situation for patients with CKD who are already faced with limited dietary choices due to their condition [40,41]. Although nutrition counseling for patients with CKD in Kenya is guided by the National Clinical Nutrition and Dietetics Reference Manual [42] alongside facility-based clinical nutrition manuals and protocols, some diet prescriptions were based on international clinical nutrition guidelines where some food items may not be locally accessible. A number of the fruits recommended for the hemodialysis patients, such as apples and grapes are imported in Kenya, hence, expensive for the patients already grappling with the reality of compromised income due to reduced economic capacity [12,43]. Prices of commodities have also been known to be regulated by their perceived demand and necessity to the target market. Certain food items might retail expensively in particular localities when traders realize that their target customers have limited options or alternatives, a situation which poses serious challenges to the dietary compliance among patients of low economic status. Furthermore, the extensive procedure involved in the preparation of renal meals exacerbates the cost implications. Integrating economic support in interventions to improve dietary adherence among CKD patients particularly in the context of poor socio-economic status of patients would thus enhance health outcomes of patients with CKD. 

Poor dietary knowledge has been associated with sub-optimal renal diet adherence in literature [29]. Therefore, consistent information and education when implementing a diet program are key to successful management of chronic conditions [36]. In our study, conflicting messages on nutrition information contributed to some participants ignoring the information received from nutritionists. This militates against the making of appropriate dietary adjustments by the patients. Participants who felt that the nutrition information they received were contradictory to what they knew as healthy diets, were less likely to adhere to prescribed diets even when correct information was presented to them. Where patients are faced with conflicting information from health professionals, they often opted to follow the dietary advice from those they trust or perceive to be more experienced and knowledgeable or those with whom they had better personal relationship. Alternatively, some patients adopt advice that they can easily integrate in their lifestyle [16]. The apparent contradictory information observed in our study could partly be attributed to the fact that although certain elements of the renal diet remain applicable to all patients, the alterations of the diets of patients with CKD often need to be individualized, taking into account comorbidities and individual body composition [44]. Lack of consistency in delivery of information by different nutritionists decried by participants in our study suggested a gap in the competency of some nutritionists working in renal units. This underscores the importance of effective communication besides the quality of patient-clinician relationships to promote dietary adherence [16]. Conflicting messages on nutrition information on what to eat or not has also been reported in literature [43]. Providing consistent dietary advice helps to minimize confusion of recommendations and improves patients’ decision-making capabilities in both CKD and non-CKD adult populations [28,45]. Individualizing diet education for patients with CKD can lead to higher adherence rates, especially when the nutrition advice is matched with the specific needs and interests of patients [46]. Interventions aimed at boosting the patient’s understanding of the disease and the basis of its dietary management can promote positive perception and attitudes regarding the effects of dietary restrictions and enhance adherence. Since the nutritional advice should be individualized based on the patient’s biochemical parameters, it appeared most participants in this study risked developing health complications if they did not follow their prescriptions from the nutritionist. 

Personal attitude towards prescribed diets during the transition period posed a challenge to adherence to for the CKD patients. This may be more burdensome and challenging to implement for patients on hemodialysis who have to change multiple dietary components such as sodium, fluid, potassium, and phosphate or those from diverse cultural and linguistic backgrounds [28]. Many of the dietary recommendations for dialysis patients are indeed highly restrictive thereby greatly limiting the dietary options for the patients [41]..Furthermore, following particular prescriptions such as low potassium diets contravenes conventional principles of healthy diet and patients have felt lethargic, malnourished, and starved if they followed the diet as prescribed [19]. The dietary recommendations in CKD may indeed make food less appetizing resulting in diminished intake and compromised nutritional status [47]. In the initial phase of CKD diagnosis, the basis for the required dietary adjustments is not well understood by patients and many of them get intimidated and feel overwhelmed by heavy restrictions placed on their diets not to mention the sudden disruption of their longstanding individual routine life [44,48]. Patients have been reported as being angry about having to take on a new food regimen in addition to changes to their daily schedules and recreational activities imposed by CKD. The feeling of anger and denial that is often expressed by patients upon being diagnosed with CKD is a threat to dietary management of the disease. It is, however, worth noting that although the Kenya Ministry of Health launched an updated Kenya National Food Composition Tables [49] based on locally available foods in 2019, most nutritionists at health facility level were not trained on the use of this resource in diet prescriptions. The use of familiar local food items during dietary counseling sessions is likely to minimize rejection of the prescriptions and improve adherence. 

### 4.2. Strengths and Limitations of the Study

This research was the first one in Kenya to explore the patients’ and caregivers’ perceptions on adherence to dietary prescriptions in CKD. Participants had an opportunity to freely express their feelings about the dietary support received from the health facility. The findings provide an opportunity for nutritionist and other healthcare providers in the renal units to realize the need to understand the patient’s views and feelings about the dietary management of CKD. 

Readers should however be aware of and consider the following limitations while interpreting the findings from our study. First, the findings may not be generalized to children or other adult patients with CKD who are not on hemodialysis. Second, our study was on adherence to overall diet prescriptions and not on nutrient-specific dietary guidelines such as fluid restriction. Third, the study setting may have had destructions due to noise/sound from the dialysis machines occasionally affecting the quality of voice recording during data collection. This required both the interviewer and participant to project their voices. However, the interviewer also took very detailed notes to mitigate the problem. Fourth, it is also worth noting that the voice recording tools can sometimes fail during data collection. In our study, two of the USB voice recorders failed leading to loss of part of the information. The interviewer changed to a working voice recorder and repeated the interview. Content from the repeat interview were compared with the initial hand-written notes to ensure that the information was consistent. Fifth, while efforts were made to minimize interviewer bias during the research process, there is possibility that unconscious bias may have occurred.

### 4.3. Implications for Policy and Practice

Based on the findings of this study, the following recommendations are suggested for policy and practice:

- More effort needs to be invested in creating public awareness of CKD in order to foster correct perceptions, attitudes and beliefs about the disease and its management so as to enhance both medication and dietary adherence among patients as well as preventive lifestyles in the general population;
- The Ministry of Heath need to build capacities of healthcare professionals with regard to nutritional management of the CKD;
- For harmonized messaging, the Ministry of Health ought to develop national standard operating procedures as well as information, education and communication materials in renal nutrition counseling.
- There is need to sensitize all nutritionists in health facilities on the updated National Food Composition Tables to encourage utilization of locally available and accessible foods in diet prescriptions;
- Healthcare professionals should seek patients’ view on impediments to dietary adherence and support them with appropriate skills to effect necessary lifestyle adjustments. This will contribute to palatable, flexible and acceptable diet recommendations for the patients.

### 4.4. Implications for Future Research

Several research questions that need further contextual understanding emerged from this study:

- What are the key motivating factors for CKD patients to adhere to diet prescriptions that can be used as a basis for improving diet management?
- How can healthcare providers, nutritionists and family caregivers minimize barriers to adherence to diet prescriptions among adult patients with CKD?
- What are the knowledge, attitudes and practices of healthcare providers towards dietary prescriptions for CKD patients?

## 5. Conclusions

The study findings provide awareness for nutritionists and other healthcare providers about CKD patients’ and caregivers’ views and feelings concerning renal diets. Both patients and caregivers are in agreement that diet prescriptions are beneficial for health and wellbeing of the patient. However, there are mixed messages to the patients and caregivers who have challenges with management and acceptability of the prescriptions. Most of them therefore make un-informed dietary decisions that lead to consumption of unhealthy foods with negative outcomes such as metabolic waste accumulation in the patients’ bodies negating the effect of dialysis and undermining the efforts of the healthcare system in CKD management.

## 6. Declarations

### Ethics Approval and Consent to Participate

Ethical approval was obtained from Kenyatta National Hospital/University of Nairobi Ethical Review Committee (P364/05/2018) and MTRH (FAN: IREC 3138). Further approval to conduct the study was obtained from the hospital directors of KNH and MTRH. Written and informed consents were obtained from study participants prior to participation.

## Figures and Tables

**Table 1:** Sub-theme 1—illustrative quotes on “perceived health benefits”.

Sub-Theme	Illustrative Quotes
Perceived health benefits of following diet recommendations	*“When you follow the diet it becomes easier to do dialysis you don’t get complications but if you take a lot of water, taste salt, meat without following instructions you will have problems of high potassium/high … what do we call it? Sodium, and what … a certain name there” (Female Patient, 42 years, Nyamira);*
	*“I’ve seen other patients who started dialyzing long time ago they are well and when I ask them, they say they follow the renal diet” (Female Patient, 40 years, from Nandi);*
	*“There is a guy I know who has kidney failure and underwent dialysis 10 times, he was told not to take alcohol, cigarette, and he later became well. He was told if he ate what he was told to avoid, the disease would relapse; now he only comes here for review” (Male Patient, 30 years old from Siaya).*

The ( ) contains participant’s information for each illustrative quote on gender, category, whether the participant was a patient or caregiver, the age and county of origin.

**Table 2:** Sub-theme 2—illustrative quotes on “ease in implementing prescribed diet”.

Sub-Theme	Sub-Category	Illustrative Quotes
Ease in implementing prescribed diet	Diet is difficult to implement	*“You cannot cook for everyone else like that. So I have to have his own cooking pan, I cook his things and put them aside before I begin cooking for the others” (Female Caregiver, 58, Nakuru);* *“You know cooking in the house; you will not cook food together with others. So his will have to be separate. And sometimes you do not have time, so you see, you start thinking that if he was not sick you would be cooking all food at once” (Female Caregiver, 38, Uasin Gishu);* *“Sometimes I avoid visiting people because I may feel hungry and yet they have only soft drinks like soda, ‘nyama choma’ (roast meat) and to avoid explaining to them all the time what I am not supposed to eat, I just avoid going to those places” (Female Patient, 40, Nandi).*
	Implementation is easy	*“When I cook her food we can eat together” (Male Caregiver, 28, Kitui);* *“We eat together my food with other members in the family. Eeeeh, eeeh” (Male Patient, 68, Kakamega).*

The ( ) contains participant’s information for each illustrative quote on gender, category, whether the participant was a patient or caregiver, the age and county of origin.

**Table 3:** Sub-theme 3—illustrative quotes on “cost of prescribed renal diet”.

Sub-Theme	Sub-Category	Illustrative Quotes
Cost of prescribed diets	The prescribed diets are not affordable	*“We are told to take white meat but sometimes we can’t afford the white meat” (Female patient, 34, Nyeri);* *“Chicken is not easy to afford for most people, people have families so it’s not easy to afford for most of them” (Male Patient, 49, Bungoma);* *“… on the side of foods, it gets expensive, especially fruits that he is to use. Its only fruits that is expensive. I don’t see if the rest are that expensive. Because he needs to use fruits every day, and you know fruits he uses are expensive” (Female Caregiver, 38, Uasin Gishu);* *“I am also told that she should eat after every 3 h but many times it is impossible with our situation” (Female caregiver, 38, Turkana);* *“You’ll have to prepare two meals—different meals and that’s a budget above what we used to have. You see as life becomes more costly and then you’re given an extra burden, life becomes a bit difficult. It comes with a budget, yeah an extra budget” (Female Caregiver, 21).*
	The prescribed renal diets are affordable	*“My wife has her business of a salon and a shop, even me I get pension so I can afford food” (Male patient, 69, Trans Nzoia);* *“Food is not a challenge in that I am working. It is not a challenge because I can afford to draw a budget and follow that budget. The challenge is the restrictions. Yes, I have money but I cannot consume most foods” (Male Patient, 56, Kakamega).*

The ( ) contains participant’s information for each illustrative quote on gender, category, whether the participant was a patient or caregiver, the age and county of origin.

**Table 4:** Sub-theme 4—illustrative quotes on “nutritional messages”.

Sub-Theme	Sub-Category	Illustrative Quotes
Nutritional information and messages	Conflicting nutrition information and messages	*“Okay, some they come and confuse us, some they come and tell us do this, do that, another one comes and tells you this, now sometimes you get confused, you don’t get the message very well. But you know, you don’t have money to pay for nutrition counseling. Confusion, that is confusion because one person will tell you eat this, the other will tell you don’t eat” (Female patient, 34, Nyeri);* *“The nutritionist advised me before and I left everything. I left all fruits! So the nurse asked me “what is wrong with you? Nowadays your blood levels are just low! You are so white what is wrong?” and I told her “I have stopped eating fruits because I was told that fruits are bad, they will harm me.” The nurse told me “no! go ahead and eat the fruits that you were eating” (Female Patient, 27, Siaya);* *“… there is a problem because; we have many nutritionists these days. One comes here and tells you this; tomorrow another one comes, tells you something different, so you don’t understand anything. Yes, that makes me sometimes decide on my own what to do. So you can’t know if may be this one is telling you the truth, may be the other one is lying. If I eat something today and it doesn’t harm me, I will eat it the next day. That’s what I do” (Female patient, 27 Siaya).*
	Clearly understood nutrition information and messages	*“Yeah am eeh—you can’t say it’s final, because you keep learning from them, you can’t say am well versed so far except that am just observing what I was told. But in case I meet a nutritionist, I still ask questions so that am sure am doing the right thing” (Male patient, 20, Baringo);* *“Yes I understood the nutrition information I received during counselling on what to eat since am just following what they told me—I was told by the nutritionist down there” (Male patient, 68, Tharaka Nithi).*

The ( ) contains participant’s information for each illustrative quote on gender, category, whether the participant was a patient or caregiver, the age and county of origin.

**Table 5:** Sub-theme 5—illustrative quotes on “transition to new diets”.

Sub-Theme	Sub-Category	Illustrative Quotes
Transition to new diets	The transition to the new diets was difficult	*“Ummh, himself, he hated it, since there were things that he loved like taking milk, drinking lots of water, and he was denied all those, in a way he was upset I mean he was not happy because what he loved are the things he was prevented from eating” (Female Caregiver, 40, Kajiado).* *“It’s a bit hard to follow most of the diet, they are not sweet I was used to eating food with fat so getting used to it is hard and it also needs a lot of preparation you can’t eat food anywhere any time you have to prepare everything for whoever is taking the diet also this foods one cannot eat and feel full you feel hungry quickly since its light” (Male Patient, 47, Nyandarua);* *“At first it was difficult but now he has accepted. Yes. He even requested we add a little salt to his food. He could not eat cabbage without salt so he would leave it then later he would feel hungry. He used to say the food is making him lose weight. It was difficult” (Female Caregiver, 41, Uasin Gishu).*
	The transition to the new diet was easy	*“She has not had any problem since she also understands that she’s ill, and if the diet prescribed to her will make improve, she likes it.” (Male caregiver, 62, Kiambu);* *“… the doctor telling me his prescriptions, so that I live longer, I felt that I should abandon those things I used to eat before and follow the doctor’s advice” (Male patient, 65, Tharaka Nithi);* *“If I find somebody has cooked food that I cannot eat I usually tell them I can’t eat it” (Female Patient, 50, Muranga).*

The ( ) contains participant’s information for each illustrative quote on gender, category, whether the participant was a patient or caregiver, the age and county of origin.

**Table 6:** Sub-theme 6—illustrative quotes on “fear of health complications and severity of disease”.

Sub-Theme	Illustrative Quotes
Fear of health complications and severity of disease in case of non-adherence to the diet prescriptions.	*“This food is not good at all, we just eat it to live” (Female Patient, 62, Nyeri);* *“I have told you since I was counseled to follow the instructions, but before I would eat a lot there would be a lot of filth building up and when I am tested it would be high. I couldn’t walk properly, I was dizzy, vomiting but since I started following the doctor’s instructions nowadays am doing well. Now I know what I am supposed to eat and what I am not meant to eat even in fruits it’s like that” (Female Patient, 30, Siaya);* *“When he eats the wrong food, he becomes sick all over again. Their bodies are very sensitive and should not eat any food. Like another day he ate something he became sick the whole night we almost woke up to bring him to hospital because he ate something he was not supposed to eat” (Male Caregiver, 22, Muranga).*

The ( ) contains participant’s information for each illustrative quote on gender, category, whether the participant was a patient or caregiver, the age and county of origin.

## Data Availability

Data is available upon request from the corresponding author at roseopiyo@uonbi.ac.ke.
